# Aspirin-triggered resolvin D1 attenuates PDGF-induced vascular smooth muscle cell migration via the cyclic adenosine monophosphate/protein kinase A (cAMP/PKA) pathway

**DOI:** 10.1371/journal.pone.0174936

**Published:** 2017-03-31

**Authors:** Giorgio Mottola, Anuran Chatterjee, Bian Wu, Mian Chen, Michael S. Conte

**Affiliations:** Department of Surgery, Division of Vascular and Endovascular Surgery, Cardiovascular Research Institute, University of California San Francisco, San Francisco, California, United States of America; Qatar University College of Health Sciences, QATAR

## Abstract

**Background and objectives:**

Resolvin D1 (RvD1) is a specialized pro-resolving lipid mediator that has been previously shown to attenuate vascular smooth muscle cell (VSMC) migration, a key process in the development of intimal hyperplasia. We sought to investigate the role of the cAMP/PKA pathway in mediating the effects of the aspirin-triggered epimer 17R-RvD1 (AT-RvD1) on VSMC migration.

**Methods:**

VSMCs were harvested from human saphenous veins. VSMCs were analyzed for intracellular cAMP levels and PKA activity after exposure to AT-RvD1. Platelet-derived growth factor (PDGF)-induced migration and cytoskeletal changes in VSMCs were observed through scratch, Transwell, and cell shape assays in the presence or absence of a PKA inhibitor (Rp-8-Br-cAMP). Further investigation of the pathways involved in AT-RvD1 signaling was performed by measuring Rac1 activity, vasodilator stimulated phosphoprotein (VASP) phosphorylation and paxillin translocation. Finally, we examined the role of RvD1 receptors (GPR32 and ALX/FPR2) in AT-RvD1 induced effects on VSMC migration and PKA activity.

**Results:**

Treatment with AT-RvD1 induced a significant increase in cAMP levels and PKA activity in VSMCs at 5 minutes and 30 minutes, respectively. AT-RvD1 attenuated PDGF-induced VSMC migration and cytoskeletal rearrangements. These effects were attenuated by the PKA inhibitor Rp-8-Br-cAMP, suggesting cAMP/PKA involvement. Treatment of VSMC with AT-RvD1 inhibited PDGF-stimulated Rac1 activity, increased VASP phosphorylation, and attenuated paxillin localization to focal adhesions; these effects were negated by the addition of Rp-8-Br-cAMP. The effects of AT-RvD1 on VSMC migration and PKA activity were attenuated by blocking ALX/FPR2, suggesting an important role of this G-protein coupled receptor.

**Conclusions:**

Our results suggest that AT-RvD1 attenuates PDGF-induced VSMC migration via ALX/FPR2 and cAMP/PKA. Interference with Rac1, VASP and paxillin function appear to mediate the downstream effects of AT-RvD1 on VSMC migration.

## Introduction

Peripheral artery disease (PAD) affects >200 million people globally, and it currently represents a major cause of morbidity as well as healthcare expenditures in the United States. Open surgery and endovascular interventions are capable of improving circulation in PAD, but their long-term efficacy is greatly diminished by excessive scarring of vessels (restenosis) which occurs in approximately half of successfully treated patients [1–9]. Intimal hyperplasia (IH) is a prototypic response to vascular injury that, when excessive, leads to restenosis [10,11]. Vascular smooth muscle cells (VSMC) and their phenotypic alterations are central to the pathophysiology of IH. VSMCs naturally reside in the tunica media; however, in response to vascular injury they migrate into the tunica intima and proliferate, causing thickening of the vessel wall and narrowing of the lumen [12,13]. Several physical and biological factors have been characterized as promoters of VSMC migration, including blood pressure, sheer stress and a vast array of cytokines and growth factors, such as the platelet-derived growth factor (PDGF) [14–16].

Resolvins constitute a family of specialized pro-resolving mediators (SPMs) derived from the omega-3 polyunsaturated fatty acids docosahexaenoic acid (DHA) and eicosapentaenoic acid (EPA). Both D-series (derived from DHA) and E-series (derived from EPA) have been shown to mediate active resolution of inflammation across a broad range of diseases (e.g. colitis, lung injury, periodontitis, etc.), and have reached the stage of early clinical trials [17–19]. In prior studies, we identified a potential “resolution-deficit” in PAD patients [20], and have shown that SPMs can modulate vascular injury responses [21–24]. Resolvin D1 (RvD1), like other members of the D-series resolvins, has two different isoforms identified in biologic samples [25,26]: 17S-resolvin D1 (17S-RvD1) and the “aspirin-triggered” 17R-resolvin D1 (17R-RvD1 or AT-RvD1). The first reaction of resolvin D1 synthesis determines whether DHA will eventually convert to the 17S or 17R isoform; 15-Lipoxygenase (15-LOX) is responsible for the synthesis of 17S-RvD1, whereas Cyclooxygenase 2 (COX-2), after being covalently modified by aspirin, promotes the synthesis of AT-RvD1. AT-RvD1 can also be produced endogenously in the absence of aspirin by alternative pathways involving cytochrome P450 enzymes [17]. The “aspirin-triggered” versions of SPMs are of particular relevance because of the ubiquitous use of aspirin for primary and secondary prevention in cardiovascular patients, identification of these mediators in human blood following fish oil supplementation [26], and their relative resistance to metabolic inactivation in comparison to the S-epimers [19,27]. Both isoforms of RvD1 bind to two known G-protein coupled receptors (GPCR), GPR32 and ALX/FPR2 [28].

In recent studies, RvD1, as well as other SPMs, was shown to attenuate VSMC migration *in vitro* as well as IH *in vivo* in murine and rabbit models of arterial injury [21,22,24]. Little is known about RvD1 signaling pathways, particularly in vascular cells. VSMC migration is a complex process involving an orchestrated concert of molecular events leading to actin polymerization, focal adhesion formation and cellular contraction [29]. Cyclic adenosine monophosphate (cAMP) and its target protein kinase A (PKA) are key mediators of cell migration in many cell types and have been extensively shown to be anti-motogenic and anti-inflammatory in VSMCs [23,30–34]. Therefore, the cAMP/PKA pathway is highly relevant to investigations of VSMC migration. In this study we demonstrate that AT-RvD1 exerts anti-motogenic effects on VSMCs via ALX/FPR2 and involving the cAMP/PKA pathway.

## Materials and methods

### Cell culture and reagents

Primary VSMCs were isolated from human great saphenous veins discarded at the time of bypass operation under a University of California San Francisco Institutional Review Board-approved protocol (UCSF Committee on Human Research- Number: 10–03395; the committee waived the need for informed consent) as described previously [35]. VSMCs were cultured at 37°C and 5% CO_2_ in low glucose Dulbecco’s modified Eagle’s medium (DMEM; HyClone Laboratories, Logan, UT, USA) containing 10% FBS (Life Technologies, Grand Island, NY). VSMCs between passages 2 and 5 were utilized for all experiments. AT-RvD1 (7S,8R,17R-trihydroxy-4Z,9E,11E,13Z,15E19Z-docosahexaenoic acid), 17S-RvD1 (7S,8R,17S-trihydroxy-4Z,9E,11E,13Z,15E,19Z-docosahexaenoic acid) were obtained from Cayman Chemical (Ann Arbor, MI, USA). The anti-GPR32 antibody was purchased from GeneTex (Irvine, CA, USA) and the anti-ALX antibody from Abcam (Cambridge, United Kingdom). PDGF-BB was purchased from Sigma-Aldrich (St. Louis, MO, USA) and Rp-8-Br-cAMP from Enzo Life Sciences (Farmingdale, NY, USA). Phorbol 12,13-dibutyrate (PDBu) was purchased from Biolog (Hayward, CA, USA) and chelerythrine chloride from Sigma-Aldrich (St. Louis, MO, USA).

### Cell migration

Migration was studied using both a scratch assay and a Transwell assay. For the scratch assay, cells were grown to near-confluence in 24-well plates, then serum-starved overnight in DMEM containing 0.1% FBS. A scratch was applied to the cell monolayer using a sterile 200μl pipette tip, and cells were pre-treated with AT-RvD1 (10nM) or vehicle (ethanol) with or without Rp-8-Br-cAMP (10μM), anti-GPR32 antibody (2μg/ml), anti-ALX antibody (2μg/ml), PDBu (10nM) or Chelerythrine (10μM) for 30 minutes. PDGF-BB (10ng/ml) was then added to the appropriate experimental groups, and initial photomicrographs were taken using a phase-contrast microscope (EVOS xl core, AMG, Mill Creek, WA). After overnight incubation, a second set of images was obtained to measure VSMC migration over the scratched area. The cell-free area for each well and time-point was measured using the software ImageJ (version 1.48, NIH; http://imagej.nih.gov/ij/download.html); we calculated the mean percent wound closure and the fold wound closure of each treatment group vs. the negative control group.

For the Transwell assay, cells were serum-starved overnight in DMEM containing 0.1% FBS. VSMC migration was assayed using 8μm-pore gelatin-coated Transwell inserts (Costar, Corning, NY). After serum starvation cells were seeded on each insert (40,000 per upper well). Cells were pre-treated with AT-RvD1 (10nM) or vehicle (ethanol) with or without Rp-8-Br-cAMP (10μM) for 30 minutes. PDGF-BB (10ng/ml) was then added to the lower well of the appropriate experimental groups and cells were allowed to incubate for 4 hours. After scraping off the non-migrated cells from the upper aspect of the porous membrane with a cotton swab, the migrated cells on the lower aspect were fixed in methanol, DAPI-stained, mounted on slides and visualized under a fluorescence microscope (EVOS fl, AMG, Mill Creek, WA). Four images of pre-selected areas (kept consistent between groups) were taken with the same camera settings for all treatment groups. After setting the same particle size threshold for all the groups, particles were automatically counted in each picture using the software ImageJ. The total of migrated cells for each sample was used to calculate the fold cell transmigration of each treatment group vs. the negative control (vehicle) group.

### cAMP measurement

Confluent VSMCs grown in 12-well plates were serum-starved overnight in DMEM containing 0.1% FBS. Cells were treated with AT-RvD1 (10nM) and harvested at different times using a 0.1N HCl, 1% Triton X-100 lysis buffer. After spinning the lysates at 12,000g for 10 minutes and collecting the supernatants, cAMP and total protein contents were measured via ELISA (Enzo Life Sciences, Farmingdale, NY, USA) and BCA assay (Thermo Fisher Scientific, Waltham, MA, USA), respectively, following the manufacturers’ protocols. cAMP concentrations in the lysates were calculated from the absorbance readouts using a four-parameter logistic fit and then normalized by total protein content.

### PKA activity assay

Confluent VSMCs grown in 24-well plates were serum-starved overnight in DMEM containing 0.1% FBS. Cells were treated with only AT-RvD1 (10nM) with or without anti-GPR32 antibody (2μg/ml) and/or anti-ALX antibody (2μg/ml). PKA activity was analyzed in crude cell lysates (2.5μg) following the protocol of a commercially available assay kit (Enzo Life Sciences, Farmingdale, NY, USA).

### Cell shape measurement

Cells were seeded on 8-well chamber slides at a density of 4,000–6,000 cells/well in DMEM containing 10% FBS for 24 hours, then serum-starved overnight in DMEM containing 0.1% FBS. Cells were pre-treated with AT-RvD1 (10nM) or vehicle (ethanol) with or without Rp-8-Br-cAMP (10μM) for 2 hours, followed by the addition of PDGF-BB (10ng/ml) for 1 hour. Cells were then fixed in 3.7% formaldehyde, permeabilized in 0.1% Triton X-100, stained with Alexa Fluor 568 phalloidin and mounted with a DAPI-containing mounting medium (Southern Biotech, Birmingham, AL, USA). One wash with PBS was performed in-between steps. Images (10x) from five pre-selected areas (consistent between groups) per well were taken using a fluorescence microscope and camera (model BX51 and DP70, Olympus, Shinjuku, Tokyo, Japan), and length to width ratios were measured for each cell (≥20 per well) using the software ImageJ.

### Rac1 activity assay

Cells were grown in 60mm dishes, and then serum starved for 24 hours. Cells were then treated with PDGF-BB (10ng/ml) and harvested at different time points for the time-course experiment. In other experiments, cells were pre-treated with AT-RvD1 (10nM) or vehicle (ethanol), with or without Rp-8-Br-cAMP (10μM) for 30 minutes, followed by PDGF-BB (10ng/ml) for 15 minutes, after which cells were lysed and immediately snap-frozen in liquid nitrogen. Rac1-GTP and total protein content were then measured using the G-LISA Rac1 Activation Assay Kit (Cytoskeleton, Inc. Denver CO, USA) following the manufacturer’s instructions. Samples were diluted in lysis buffer to normalize total protein concentrations before Rac1-GTP measurement. Results were calculated from the absorbance readouts, and are shown as fold change vs. the negative control.

### Western blotting (phospho-VASP detection)

Cells were grown to near-confluence in 60mm dishes, and than serum starved for 48 hours. In a preliminary set of experiments, cells were treated with AT-RvD1 (10nM) and harvested at different time points. In subsequent experiments cells were pre-treated with AT-RvD1 (10nM) or vehicle (ethanol) with or without Rp-8-Br-cAMP (10μM) for 30 minutes, followed by PDGF-BB (10ng/ml) treatment for 5 min. Cells were then lysed using CellLytic M lysis buffer (Sigma-Aldrich, St. Louis, MO, USA) plus Halt Protease Inhibitor Cocktail (Thermo Fisher Scientific, Waltham, MA, USA) and immediately snap-frozen in liquid nitrogen. After constant agitation at 4°C for 30min, samples were centrifuged at 12,000g for 20 minutes, and the supernatants (whole cell extracts) were collected. Total protein concentration was assessed using the BCA assay (Thermo Fisher Scientific, Waltham, MA, USA) and 30μl of each protein sample was loaded on 4–12% gradient electrophoresis gels (Thermo Fisher Scientific, Waltham, MA, USA) and run at 200V for 22 minutes. A protein wet transfer on PVDF membrane was then performed at 4°C (100V for 60 minutes) followed by blocking the membranes in 5% non-fat dry-milk with Tris-Buffered Saline and Tween-20 (TBST) for 60 minutes. The membranes were probed with anti-p-VASP Ser 157 (1:1000 dilution, Cell Signaling Technologies, Danvers, MA, USA) and anti-β-Actin (Sigma-Aldrich, St. Louis, MO, USA) antibodies at 4°C overnight. The next day membranes were washed, probed with an HRP-conjugated antibody (Santa Cruz Biotechnologies, Santa Cruz, CA, USA), and bands were visualized through chemiluminescence using the WesternBright quantum kit (Advansta, Menlo Park, CA, USA). Blots were visualized using the ChemiDoc MP system (Bio-Rad, Hercules, CA, USA) and bands analyzed using the ImageLab (version 4.0, Bio-Rad, Hercules, CA, USA). Results are shown as fold-changes vs. time 0 or negative control of band intensity ratios between p-VASP and β-Actin.

### Immunofluorescent staining (paxillin)

Cells were seeded in 4-well chamber slides, and then serum-starved overnight in DMEM containing 0.1% FBS. A scratch assay was performed as described above. After cell migration occurred overnight, cells were fixed with 3.7% formaldehyde, permeabilized with 0.5% Triton X-100, blocked with 2% Bovine Serum Albumin + 0.3% Triton X-100, and incubated with anti-paxillin primary antibody (1:50 dilution, Santa Cruz Biotechnology, Santa Cruz, CA, USA) overnight at 4°C. After that, cells were incubated with Alexa Fluor 488 secondary antibody (Thermo Fisher Scientific, Waltham, MA, USA) at room temperature for 1 hour. Three washes with PBS were performed in-between steps. Cells were then mounted with a DAPI-containing mounting medium (Southern Biotech, Birmingham, AL, USA). Five images of pre-selected areas at the edge of the scratch (kept consistent between groups) were taken with the same camera settings for all treatment groups using a fluorescence microscope and camera (model BX51 and DP70, Olympus, Shinjuku, Tokyo, Japan). After setting the same particle size threshold for all the groups, particles were automatically counted within the cells in each picture using the software ImageJ and normalized by the number of DAPI-stained nuclei in the same picture. Results are shown as fold change vs. the negative control group.

### Statistical analysis

Data is shown as mean ± standard error of mean (SEM). One-way Analysis of Variance (ANOVA) was run first on all the treatments groups, followed by Sidak’s or Dunnett’s *post hoc* tests for multiple comparisons. For all analyses, P = 0.05 was considered to be the threshold for statistical significance.

## Results

### AT-RvD1 and 17S-RvD1 attenuate PDGF-induced VSMC migration

**Fig 1:**
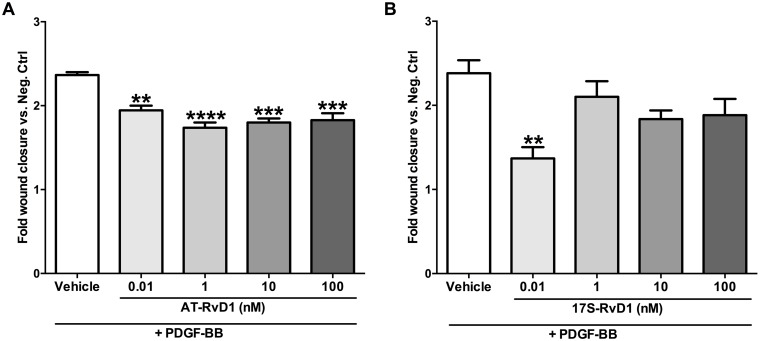
RvD1 epimers attenuate PDGF-induced migration in VSMCs. AT-RvD1 inhibits the VSMC migratory response induced by PDGF-BB (10ng/ml) in a scratch assay within a dose range of 0.01–100nM (**A**, n = 3). 17S-RvD1 also showed a significant reduction in VSMC migration at the lowest nanomolar dose (0.01nM; **B**, n = 3). **P<0.01 vs. positive control, ***P<0.001 vs. positive control, ****P<0.0001 vs. positive control.

We compared the effects of the two isomers of RvD1 (17S and 17R) on VSMC migration. In the scratch assays, AT-RvD1 (0.01–100nM) significantly reduced VSMC migration across a wound after stimulation with PDGF-BB (Fig 1A). We did not observe an obvious dose-dependent response, but rather a trend of efficacy at the low nanomolar range (1–10nM). 17S-RvD1 also showed anti-migratory effects in the lower dose range with a statistically significant effect at 0.01nM (P<0.01; Fig 1B).

### AT-RvD1 activates the cAMP/PKA pathway in VSMCs

**Fig 2:**
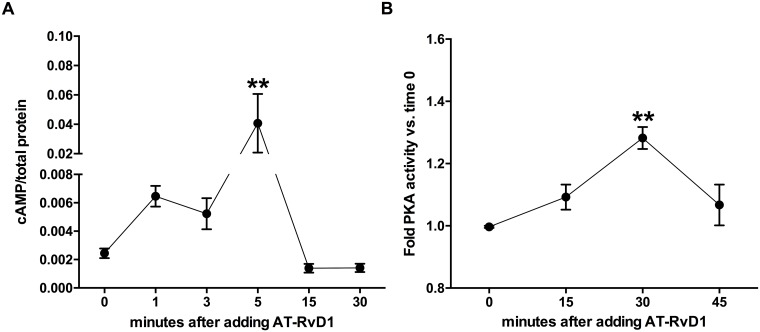
AT-RvD1 increases cAMP levels and PKA activity in VSMCs. AT-RvD1 (10nM) rapidly increases cAMP levels peaking at 5 minutes and returning to baseline at 15 minutes (**A**, n≥4). AT-RvD1 (10nM) also increases PKA activity in VSMC with peak levels seen at 30 minutes (**B**, n = 3). **P<0.01 vs. time 0.

As the PKA pathway is known to play a critical role regulating VSMC migration, we sought to investigate whether changes in cAMP levels and PKA activity could be induced by AT-RvD1. We found that AT-RvD1 causes a rapid increase in cAMP levels in VSMCs, peaking at 5 minutes (P<0.01) and subsequently returning to baseline at 15 minutes (Fig 2A). We also observed a significant increase in PKA activity induced by AT-RvD1 peaking at 30 minutes (P<0.01; Fig 2B).

### AT-RvD1 attenuates PDGF-induced VSMC migration through a cAMP/PKA-dependent mechanism

**Fig 3:**
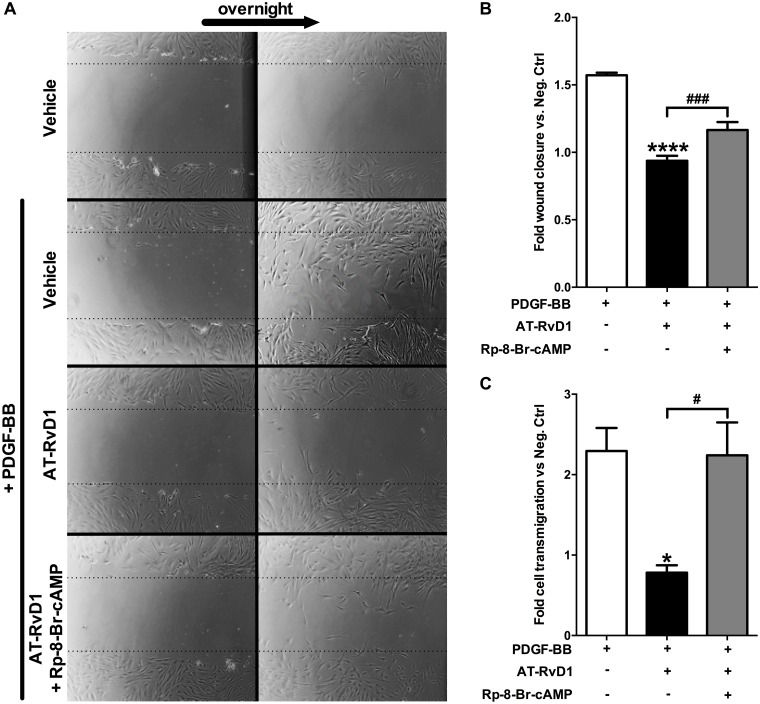
AT-RvD1 attenuates PDGF-induced migration in VSMCs by the cAMP/PKA pathway. AT-RvD1 (10nM) attenuates PDGF-induced migration in VSMC in a scratch assay and in a Transwell assay. The PKA specific inhibitor (Rp-8-Br-cAMP 10μM) partially reverts the effect of AT-RvD1 on VSMC migration in the scratch assay (**A** & **B**, n = 3) and almost completely in the Transwell assay (**C**, n = 3).

To test a possible link between the AT-RvD1 anti-migratory effect and the cAMP/PKA pathway, we performed the VSMC scratch and Transwell migration assays in the presence of AT-RvD1 and the specific PKA inhibitor Rp-8-Br-cAMP. In the scratch assay, AT-RvD1 significantly inhibited VSMC migration induced by PDGF (40% inhibition of migration; P<0.0001) and this effect was markedly attenuated by inhibition of PKA (38% reversal of AT-RvD1 effect; P<0.001), suggesting an involvement of the kinase in the mechanism of action (Fig 3**A** & 3**B**). Significantly, in the Transwell assay we observed strong inhibition of PDGF-induced migration by AT-RvD1 treatment (498 average migrated cells vs. 170; 66% inhibition of migration; P<0.05) and an almost complete reversal of this effect with inhibition of PKA (170 average migrated cells vs. 486; 97% reversal of AT-RvD1 effect; P<0.05) (Fig 3**C**). Activating or inhibiting PKC by using PDBu or Chelerythrine, respectively, did not change the anti-migratory effect of AT-RvD1 in the scratch migration assay (S1 Fig); this finding suggests that PKC does not mediate the effect of AT-RvD1 on VSMC migration.

### AT-RvD1 attenuates PDGF-induced cytoskeletal rearrangements in VSMCs via PKA activity

**Fig 4:**
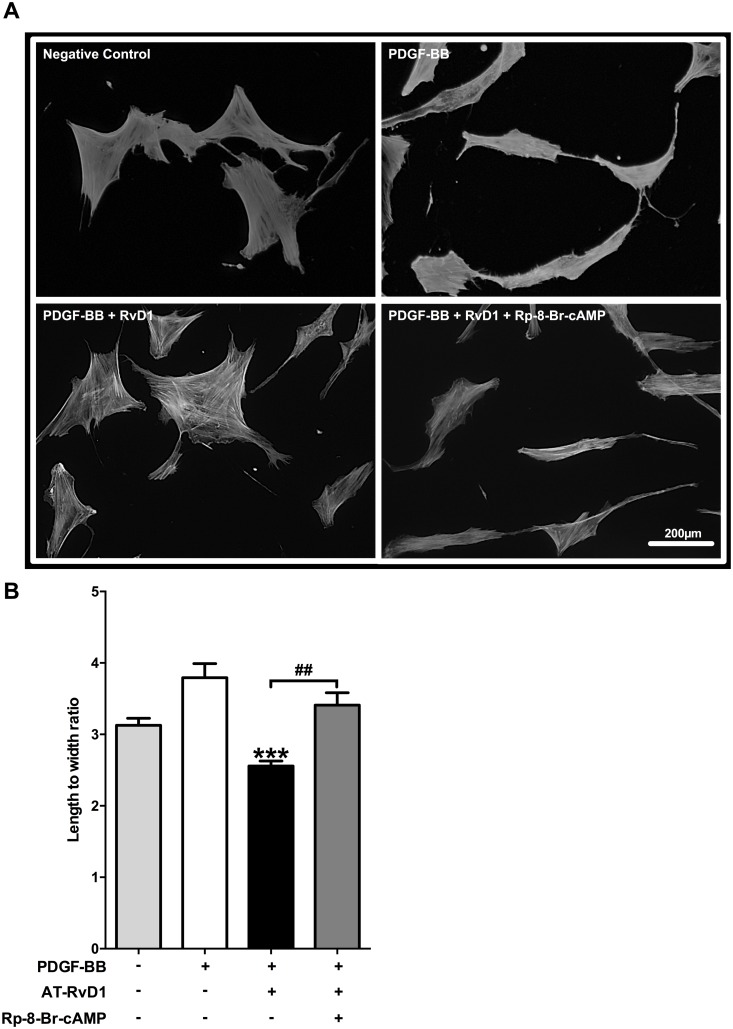
AT-RvD1 attenuates PDGF-induced cytoskeletal rearrangements in VSMCs via PKA. VSMCs were stained using an actin-phalloidin staining (representative pictures are shown in panel **A**). PDGF-BB (10ng/ml) caused a significant increase of VSMC length to width ratio which reflects pro-migratory cytoskeletal rearrangements (P<0.05 vs. negative control). AT-RvD1 (10nM) reduced this effect by lowering the VSMC length to width ratio below the value observed in the negative control. The PKA specific inhibitor (Rp-8-Br-cAMP 10μM) reversed the effects of AT-RvD1. (**B**, n = 4). ***P<0.001 vs. positive control, ^##^P<0.01.

We then turned our attention towards downstream pathways to investigate potential mechanisms of action. PDGF is known to rapidly increase VSMC length to width ratio through stress fiber formation in the initiation of a migratory response. We found that AT-RvD1 reduced the length to width ratio by 32% compared to PDGF-BB only (P<0.001) and that the inhibition of PKA with Rp-8-Br-cAMP reversed this cytoskeletal effect (P<0.01; Fig 4).

### AT-RvD1 interferes with PDGF-induced Rac1 activation via PKA

**Fig 5:**
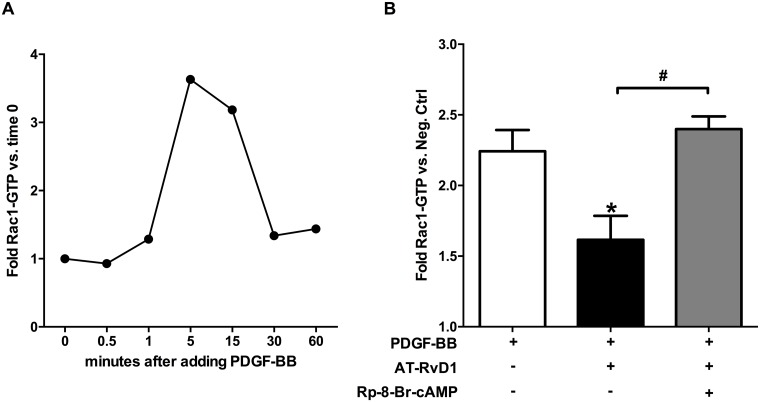
AT-RvD1 attenuates PDGF-induced Rac1 activation via PKA. PDGF-BB (10ng/ml) stimulation of VSMCs induces a rapid Rac1 activation peaking at 5–15 minutes and dropping at 30 minutes (**A**; n = 1). AT-RvD1 (10nM) attenuates Rac1 activation induced by PDGF-BB (10ng/ml) at 15 minutes; the effect of AT-RvD1 is abrogated by adding a PKA specific inhibitor (Rp-8-Br-cAMP 10μM)(**B**; n≥4).

Actin polymerization dynamics are central to the cytoskeletal rearrangements that take place during VSMC leading edge formation and migration. Rac1 is a crucial regulator of actin filament formation, and its activity is required to promote VSMC migration [36]. Since PDGF-BB is known to activate Rac1, we investigated this key protein as a downstream target candidate of AT-RvD1 signaling. As the active form of Rac1 (Rac1-GTP) is a labile entity and prone to hydrolysis to its inactive form (Rac1-GDP), we conducted a preliminary time-course experiment to characterize Rac1 activation dynamics in PDGF-stimulated human VSMCs. Rac1-GTP levels increased rapidly and peaked around 5–15 minutes after PDGF-BB addition, returning to baseline by 30 minutes (Fig 5A). Based on these results, we tested the effect of AT-RvD1 (10nM) at 15 minutes after PDGF-BB stimulation. We found that AT-RvD1 decreased the active form of Rac1 (Rac1-GTP) in VSMCs by 50% compared to the positive control (PDGF-BB only; P<0.05), an effect that was abolished by PKA inhibition (P<0.05; Fig 5B).

### AT-RvD1 induces VASP phosphorylation via PKA

**Fig 6:**
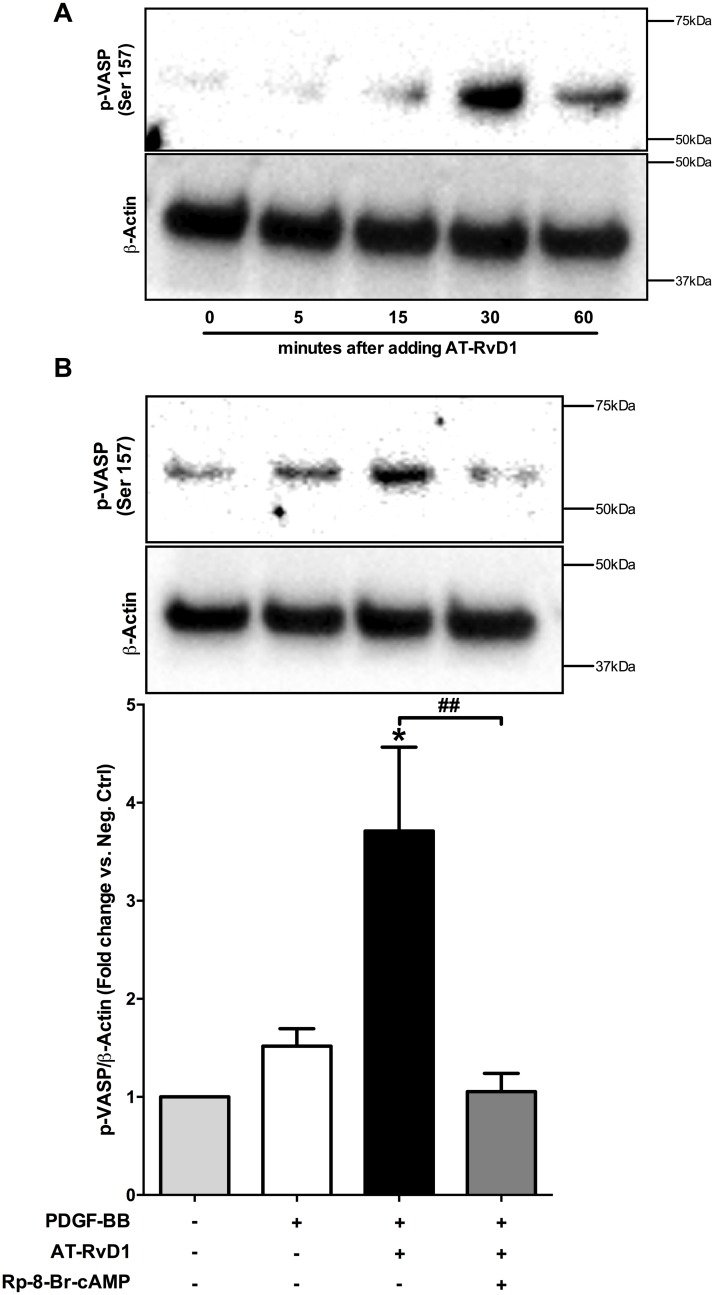
AT-RvD1 induces VASP phosphorylation via PKA. AT-RvD1 alone induced VASP phosphorylation which peaked at 30 minutes as shown in a representative blot (**A**; n = 2). PDGF-BB alone did not change VASP phosphorylation significantly vs. negative control. AT-RvD1 (10nM) induced VASP phosphorylation in the presence of PDGF-BB as shown in a representative blot and quantification of three independent experiments. The induction of phosphorylation was abolished by addition of a PKA specific inhibitor (Rp-8-Br-cAMP 10μM)(**B**; n = 3). *P<0.05 vs. positive control, ^#^P<0.05, ^##^P<0.01.

VASP is another crucial regulator of actin polymerization, which down-regulates actin filament formation in its phosphorylated form [37]. Since PKA is known to phosphorylate VASP [30,37] we sought to investigate whether AT-RvD1 could be involved in this event. In two independent experiments AT-RvD1 (10nM) increased VASP phosphorylation in VSMC, with a maximal change at 30 minutes (Fig 6A). In the presence of PDGF-BB, VASP phosphorylation was also increased in AT-RvD1-treated cells when compared to the negative control and cells treated with PDGF-BB only (P<0.05). Cells treated with PDGF-BB only did not show significant changes in VASP phosphorylation compared to negative control. As expected, inhibiting PKA activity effectively abolished AT-RvD1 induction of VASP phosphorylation (P<0.01; Fig 6B).

### AT-RvD1 interferes with paxillin localization to focal adhesion via PKA

**Fig 7:**
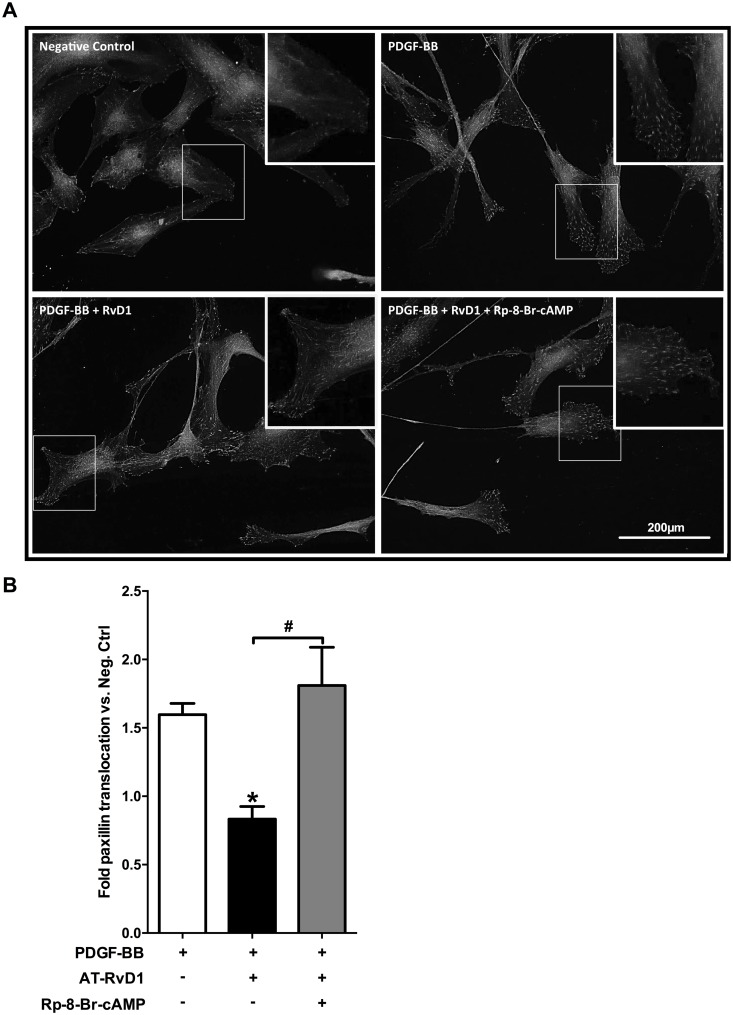
AT-RvD1 prevents paxillin localization to focal adhesions in PDGF-stimulated VSMCs. Total paxillin was visualized in VSMCs using immunofluorescence (representative pictures are shown in panel **A**). By quantifying the number of particles (normalized by cell number) we observed that PDGF-BB (10ng/ml) induced localization of paxillin to focal adhesions at the leading edge of migrating VSMCs, while AT-RvD1 (10nM) attenuated this effect. PKA inhibition (with Rp-8-Br-cAMP 10μM) reversed the AT-RvD1 effect significantly (**B**; n = 4). *P<0.05 vs. positive control, ^#^P<0.05.

Focal adhesions act in concert with actin polymerization during migration, making their contribution critical to cellular motility. Since paxillin is one of the most important constituents of focal adhesion complexes and participates downstream of PDGF signaling, we tested the effects of AT-RvD1 in modulating the intracellular localization of paxillin in migrating VSMCs. We found that AT-RvD1 reduced paxillin localization in focal adhesions of migrating VSMC in a scratch assay (Fig 7) compared to the positive control group (P<0.05). Furthermore, PKA inhibition reversed the effects of AT-RvD1 on paxillin localization (P<0.05).

### AT-RvD1 modulates PKA activity and cell migration in VSMCs through ALX/FPR2

**Fig 8:**
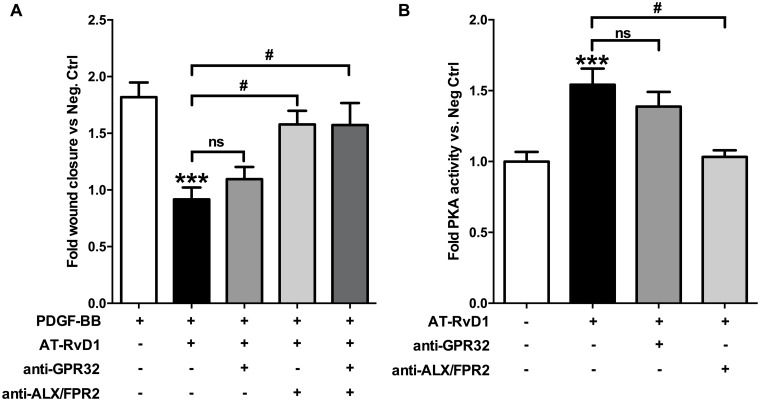
AT-RvD1 effects on VSMC migration and cAMP levels are mediated by ALX/FPR2. AT-RvD1 (10nM) reduced PDGF-BB-induced (10ng/ml) VSMC migration in a scratch assay, and blocking ALX/FPR2 with a specific antibody (2μg/ml) neutralized this effect whereas an anti-GPR32 specific antibody (2μg/ml) showed virtually no effect (**A**, n≥3). The AT-RvD1-induced increase in PKA activity at 30 minutes is almost completely reversed by an anti-ALX/FPR2 specific antibody (2μg/ml) whereas an anti-GPR32 specific antibody (2μg/ml) showed only a not statistically significant partial reversal (**B**; n = 6). ***P<0.001 vs. positive control, ^#^P<0.05.

RvD1 functions through at least two known G protein-coupled receptors, GPR32 and FPR2/ALX; We observed that by blocking ALX/FPR2 we almost completely reversed AT-RvD1 effects on PDGF-stimulated migration (Fig 8A). Inhibition of ALX/FPR2 also completely abolished AT-RvD1 induced up-regulation of PKA activity at 30 minutes (P<0.05), while after blocking GPR32 we observed only a partial and not statistically significant reversal of the effect (Fig 8B).

## Discussion and conclusions

**Fig 9:**
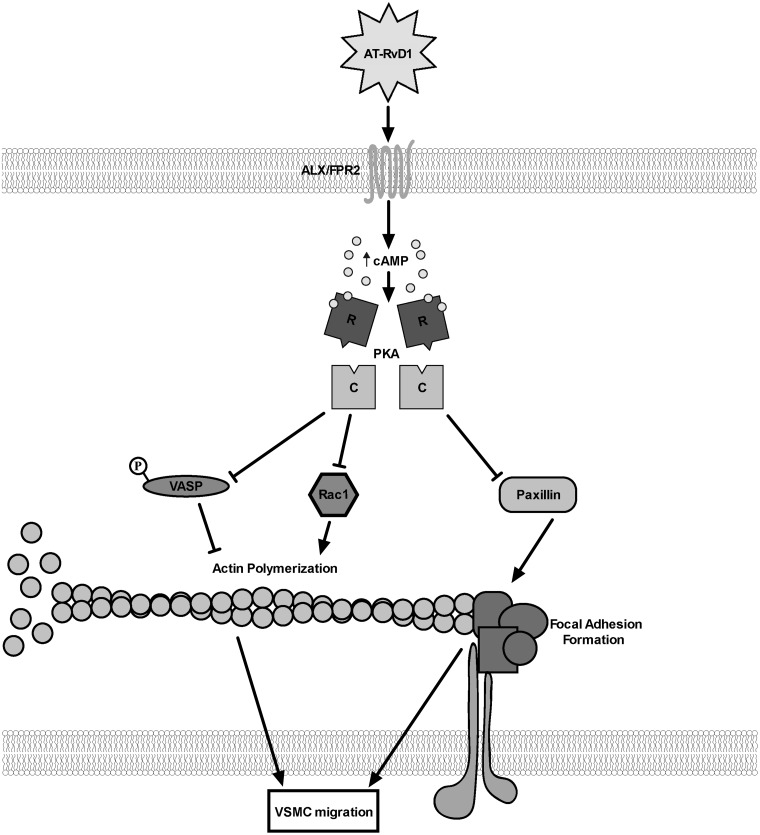
Schematic of proposed pathway by which RvD1 modulates VSMC migration. The schematic shows a summary of our findings in a proposed simplified mechanism for the effects of AT-RvD1 on VSMC migration. AT-RvD1 increases cAMP levels at least by activating ALX/FPR2; the subsequent activation of PKA interferes with actin polymerization by inhibiting VASP (by phosphorylation) and Rac1, and with focal adhesion formation by decreasing paxillin localization to the cell leading edge. “R” and “C” represent the regulatory and the catalytic subunits of PKA, respectively.

VSMC migration is a critical feature of the prototypic response to vascular injury, and an essential step in IH development and restenosis. Recent studies have demonstrated a range of vasculo-protective properties of the DHA-derived SPM [21,22,24], including inhibition of VSMC migration; however the underlying molecular mechanisms are unclear. 17R-RvD1 is the aspirin-triggered isoform of RvD1, making it particularly of interest clinically as most patients affected by atherosclerosis receive long-term aspirin treatment. In a recent report, AT-RvD1 was detected by liquid chromatography–tandem mass spectrometry (LC-MS/MS)-based metabololipidomics in human plasma from patients affected by coronary heart disease treated with aspirin with and without an EPA and DHA supplement [26]. AT-RvD1 released from a bio-degradable material has been recently shown to decrease neutrophil infiltration and to increase the anti-inflammatory monocyte/macrophage population in a murine model [38]. In another recent study, AT-RvD1 has been shown to suppress TGF-β-induced migration and invasion of A549 lung cancer cells [39]. In the present study, we investigated possible mechanisms through which AT-RvD1 exerts an anti-migratory effect in VSMCs. PDGF, among other trophic factors and cytokines, is established as a potent migratory stimulus in acute vascular injury. Our findings suggest that ALX/FPR2, via the second messenger cAMP and its downstream target PKA, is involved in AT-RvD1 modulation of VSMC phenotype in the presence of PDGF-BB. In addition, we demonstrate that AT-RvD1 signaling directly interferes with key components of the actin polymerization machinery and focal adhesion formation in VSMCs, and that these effects are also dependent, at least in part, on the cAMP/PKA pathway (Fig 9). Collectively, these findings add new insight into the mechanisms by which RvD1 exerts direct effects on VSMC phenotype.

The mechanism of action of SPMs and particularly of resolvins remains greatly unexplored in vascular cells, with most current knowledge based on studies in leukocytes and other non-vascular cell types [40]. SPMs, like other locally acting autacoids, are likely to exert differential cell- and context-dependent effects. In neutrophils, RvE1 attenuates TNF-α signaling through ERK activation, and blocks cAMP production and NF-κB signaling by binding to its receptors ChemR23 and BLT1, respectively [41]. In macrophages, RvD1 altered specific miRNAs involved with resolution signaling, in a receptor-dependent fashion [42]. In lung cancer cells, AT-RvD1 has been recently shown to attenuate TGF-β1-induced epithelial-to-mesenchymal transition by inhibiting the mTOR pathway [39]. In this study, we report the involvement of cAMP in mediating AT-RvD1 effects in VSMCs. Interestingly, cAMP has been recently shown to be involved in RvD1 signaling in other cell types; in one study, RvD1 increased alveolar fluid clearance by activating the Na, K-ATPase channel via the ALX/cAMP/PI3K pathway [43], and in another study it was found to decrease MK2 phosphorylation via cAMP in bone marrow-derived macrophages [44]. Although the cAMP pathway regulates a wide variety of cellular functions, its role in cell motility is widely appreciated in multiple cell types [30]. cAMP has consistently emerged as an important negative regulator of VSMC migration, and at least part of this activity appears as a consequence of interference with the PDGF signaling pathway. For example, the β-adrenergic receptors are well known down-regulators of PDGF-induced VSMC migration, and they act by elevating intracellular cAMP levels [45,46]. Moreover, a recent study showed that cAMP-phosphodiesterase 1C regulates VSMC growth, migration and IH, suggesting a protective role of cAMP in VSMC proliferation [47]. While the present study did not examine proliferative pathways, we have previously demonstrated cytostatic activity of D-series resolvins in VSMC *in vitro* and *in vivo* [21,24].

A recent study showed elevation of cAMP in murine macrophages by RvD1 [48], and our prior studies with the DHA-derived SPM maresin-1 (Mar-1) showed a similar effect of increasing intracellular cAMP in human VSMCs and endothelial cells [23]. In this study we observed an acute, transient increase in cAMP levels in VSMCs following exposure to AT-RvD1; by 15 minutes cAMP levels returned to baseline levels. Concurrently, we observed a significant increase in PKA activity induced by AT-RvD1 30 minutes after administration that was almost completely abolished by blocking ALX/FPR2. Our data demonstrates a link between the cAMP/PKA pathway and the anti-migratory phenotypic and downstream signaling in VSMC mediated by AT-RvD1.

In addition, we identified that some of the downstream targets of PKA known to be involved in cell migration are altered by AT-RvD1 in a PKA-dependent fashion. Rac1 is a small G-protein that has been characterized extensively as a critical regulator of the actin polymerization machinery [49,50]. Rac1 activity promotes the formation of actin filaments and has been shown to be required for PDGF-induced fibroblast and VSMC migration [36]. Although PKA has been shown to interact with Rac1, the exact relationship between the two proteins remain largely unknown and seem to vary considerably between different cell types [30]. A recent study has shown that Exendin-4 (a glucagon-like protein-1 receptor agonist) inhibits Rac1 activity through PKA in VSMCs which supports a negative regulation of actin filament formation by cAMP in these cells [51]. In our study, Rac1 activation by PDGF was significantly attenuated by AT-RvD1 in a PKA-dependent fashion. While the connection between PKA and VASP is well characterized in VSMCs [30,32,37,45], the one between PKA and Rac1 is complex and not well defined. For instance, PKA has been shown to directly phosphorylate Rac1 in neuronal cells and thus inhibit its activity [52]. *In vitro*, PKA also phosphorylates and thus inactivates P-Rex1, a type of GEF (Guanine nucleotide Exchange Factor) involved in the activation of Rac1 [53,54]. Interestingly, in a recent study, RvD1 was shown to induce phagocytosis in macrophages by increasing Rac1 activation [55]. Given the importance of actin polymerization in both phagocytosis and cell motility, and the homeostatic effects of RvD1 on efferocytosis and migration, it is not surprising that RvD1 might demonstrate opposite effects on Rac1 in different cell types and contexts.

VASP is a well-established target of PKA; via phosphorylation at serine 157, PKA diminishes the actin nucleating activity of VASP as well as its binding to actin filaments, thereby inhibiting smooth muscle migration [30,37,45]. We observed an effect of AT-RvD1 on VASP phosphorylation/inactivation, and this mechanism most likely inhibits migration independent of the PDGF pathway since the growth factor alone did not induce significant changes in phospho-VASP levels. The effects of AT-RvD1 on VASP were mediated via PKA, as Rp-8-br-cAMP completely abolished the change. To our knowledge, this is the first evidence that links AT-RvD1 to VASP phosphorylation through PKA. As shown by others, VASP phosphorylation via cAMP promoted endothelial barrier function [56]; since cAMP in general has an anti-inflammatory role in endothelial cells, the observed protective effects of Mar-1 or RvD1 in animal models of acute lung injury [43,57] could be imparted through elevation of cAMP levels. Interestingly in murine macrophages [48] and human endothelial cells (unpublished data from our lab) RvD1 has been shown to increase intracellular cAMP levels. The extent of involvement of VASP in mediating RvD1 actions under various migratory agonists remains to be explored.

Paxillin, in response to motogenic stimuli such as PDGF-BB, localizes towards the leading edges of the cell and functions as a scaffold for other proteins, including Src and FAK, to promote focal adhesion complex formation and thus migration [58]. Regulation of paxillin by PKA remains unclear in VSMCs; however, PKA has been shown to prevent paxillin localization in focal adhesions through disruption of its interactions with integrins in human leukocytes [59]. Moreover, another study demonstrated cAMP to inhibit migration in pancreatic adenocarcinoma cells, and PKA had a specific role in preventing paxillin accumulation in focal adhesions within those cells [60]. We found that AT-RvD1 interfered with the PDGF pathway by attenuating paxillin localization at the leading edge of migrating VSMCs, suggesting decreased focal adhesion formation as an important mechanism by which AT-RvD1 can regulate VSMC migration. Although AT-RvD1 effects on paxillin appeared PKA-dependent, the intermediate effectors between PKA and paxillin remains to be explored. Of note, we were unable to detect significant changes in paxillin phosphorylation levels by AT-RvD1 via immunofluorescence in VSMCs (data not shown).

The molecular mechanisms underlying RvD1 signaling in vascular cells remain a subject of ongoing investigation. Although the cAMP/PKA pathway appears to be involved in the regulation of VSMC migration by AT-RvD1, it likely represents one component within a more complex signaling cascade. We used two different migration assays to test for PKA dependency of AT-RvD1 effects on VSMC migration. PKA inhibition almost completely reversed the effects of AT-RvD1 on VSMC migration in the Transwell assay but less so in the scratch assays; the partial reversal in the scratch assay suggests that other pathways likely act in concert with the cAMP/PKA axis. Neither of the two migration assays measures migration selectively, as cell adhesion and proliferation may also be involved in the measured responses. Since PKC has been shown to promote inhibition of VSMC migration [61] we utilized a PKC activator and inhibitor in order to determine whether or not the anti-migratory effect of AT-RvD1 could be in part ascribed to PKC in addition to PKA; our results did not support an interaction between AT-RvD1 and the PKC pathway in this context (S1 Fig). At the receptor level, we observed that ALX/FPR2 was associated with both PKA activity as well as downstream anti-migratory phenotype induced by AT-RvD1 in VSMC; GPR32, on the other hand, only partially inhibited the rise in PKA activity and had minimal effect on migration. In prior studies we had observed a greater role of GPR32 compared to ALX/FPR2 in mediating the anti-migratory effects of 17S-RvD1 [21]; this discrepancy might suggest that the two isomers interact differently with these two receptors or might be attributed to inherent limitations of the blocking antibodies. Knock-down or overexpression studies might be useful to further delineate the role of GPR32 in AT-RvD1 signaling; however, these manipulations are not physiological, and are challenging to achieve in primary cultured human VSMCs. Furthermore, it is not known how ALX/FPR2 or GPR32 affects cAMP (e.g. via direct activation of adenylyl cyclase or indirectly via inhibition of phosphodiesterases), making this a relevant area of future investigation.

In summary, we demonstrate that the cAMP/PKA pathway is involved in the anti-motogenic effects of AT-RvD1 in PDGF-stimulated VSMCs. The RvD1 receptor ALX/FPR2 is strongly implicated in this pathway. AT-RvD1 exposure interferes with the actin polymerization machinery and focal adhesion formation in VSMCs. As we have previously identified bioactive SPMs and their receptors in the vessel wall [21] these findings provide potential insight into the molecular mechanisms of resolution of acute vascular injury.

## Supporting information

S1 Fig PKC does not mediate AT-RvD1 anti-migratory action in VSMCs. AT-RvD1 (10nM), the PKC activator PDBu (10nM) and the PKC inhibitor Chelerythrine (10μM) did not cause any significant change in migration compared to negative control. As expected, AT-RvD1 significantly attenuated PDGF-induced VSMC migration; however, the addition of PDBu and Chelerythrine did not change AT-RvD1. PDBu alone did show a reduction in PDGF-induced migration, but the change was not statistically significant. Chelerythrine alone had no significant effect on PDGF-induced migration (n = 3). *P<0.05 vs. positive control.
(TIF)

S1 File Data set of the study. The spreadsheet contains all data underlying the findings described in this article.
(XLSX)
