# Supplementary figures and images for: Aspirin-triggered resolvin D1 attenuates PDGF-induced vascular smooth muscle cell migration via the cyclic adenosine monophosphate/protein kinase A (cAMP/PKA) pathway
Source: PLoS One. 2017 Mar 31;12(3):e0174936. doi: 10.1371/journal.pone.0174936 (PMC5376330; doi:10.1371/journal.pone.0174936)

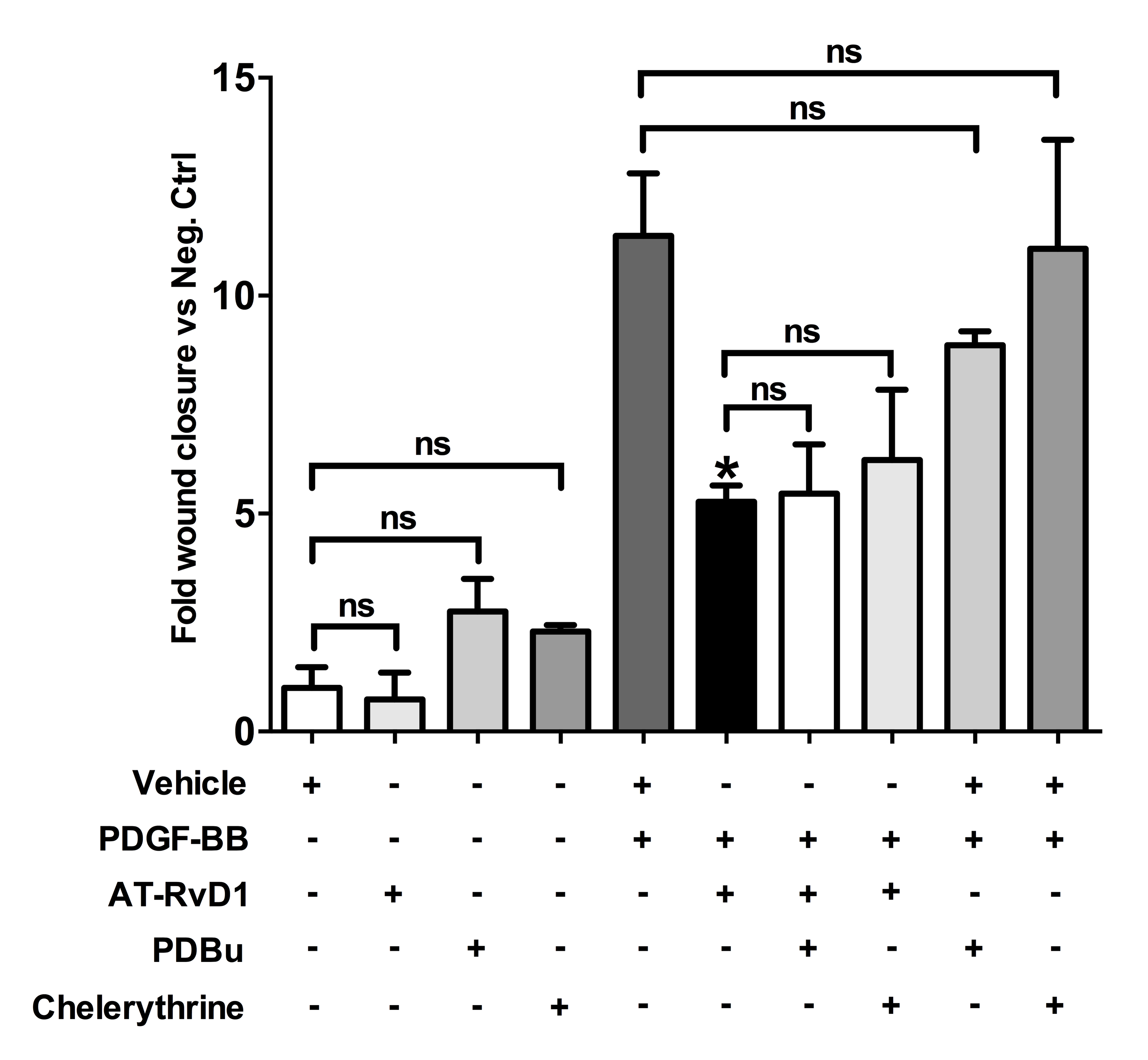

Supplement: S1 Fig — AT-RvD1 (10nM), the PKC activator PDBu (10nM) and the PKC inhibitor Chelerythrine (10μM) did not cause any significant change in migration compared to negative control. As expected, AT-RvD1 significantly attenuated PDGF-induced VSMC migration; however, the addition of PDBu and Chelerythrine did not change AT-RvD1. PDBu alone did show a reduction in PDGF-induced migration, but the change was not statistically significant. Chelerythrine alone had no significant effect on PDGF-induced migration (n = 3). *P<0.05 vs. positive control. (TIF) [file pone.0174936.s001.tif]
